# Trauma-associated sleep disturbances among women in a nationwide study

**DOI:** 10.1038/s43856-026-01698-0

**Published:** 2026-06-17

**Authors:** Anna Bara Unnarsdottir, Arna Hauksdottir, Thor Aspelund, Erla Bjornsdottir, Svava Dogg Jonsdottir, Gunnar Tomasson, Vincent Mysliwiec, Unnur Anna Valdimarsdottir, Edda Bjork Thordardottir

**Affiliations:** 1 Centre of Public Health Sciences, Faculty of Medicine, School of Health Sciences, University of Iceland, Reykjavik, Iceland; 2 Reykjavik University, Reykjavik, Iceland; 3 Department of Rheumatology, University Hospital, Reykjavik, Iceland; 4 University of Texas Health Science Center San Antonio, San Antonio, TX USA; 5 Institute of Environmental Medicine, Unit of Integrative Epidemiology, Karolinska Institutet, Stockholm, Sweden

**Keywords:** Epidemiology, Epidemiology

## Abstract

**Background:**

Trauma-associated sleep disorder is a proposed sleep disorder characterized by trauma-related nightmares, hyperarousal during sleep, and disruptive nocturnal behaviors following exposure to a traumatic event. Sleep problems are common after trauma, yet little is known about their prevalence among women in the general population. We aimed to estimate the prevalence of trauma-associated sleep disturbances (TASD) and identify associated factors in a large nationally representative cohort.

**Methods:**

We used data from a population-based cohort of Icelandic women aged 18 to 69 years. Of the 30,403 women who provided informed consent, 27,938 women were included in the analysis. Participants completed assessments of trauma history, TASD, and other mental health outcomes. Past month TASD were identified using established symptom criteria. Prevalence ratios were estimated using modified Poisson regression adjusted for demographic, socioeconomic, behavioral, and trauma-related factors.

**Results:**

Here we show that 6.9% of women in the cohort report TASD. Prevalence was highest among young women and those exposed to physical or sexual violence. Experiencing the worst life stressor more than once and close temporal proximity to the worst life stressor was also associated with higher TASD prevalence. TASD are strongly associated with symptoms of posttraumatic stress disorder (PTSD), depression, anxiety, and general sleep problems.

**Conclusions:**

Results indicate that TASD affect a substantial number of women in the general population and are most prevalent among women exposed to interpersonal violence and among those with repeated or recent traumatic experiences. These findings underscore the importance of early identification of TASD and targeted interventions in healthcare.

## Plain language summary

Some people develop sleep problems after experiencing a difficult life event. These problems can include nightmares, trouble staying asleep, acting out dreams and talking during sleep without fully awakening. We wanted to understand how common these issues are among women and which traumatic experiences are linked to them. Approximately twenty-eight thousand women took part in our study. We found that about 7 out of every 100 women had these symptoms in the past month. They were more common in women who had experienced sexual or physical violence, repeated trauma, or a recent traumatic event. Our results show that sleep can be an important sign of how someone is coping after trauma. Paying attention to sleep problems may help identify women who could benefit from support or treatment.

## Abstract (web summary)

Unnarsdottir et al. examine the prevalence and correlates of trauma-associated sleep disturbances in a nationwide cohort of 27,938 women. Findings show that nearly 7% of women report these disturbances, which are strongly associated with interpersonal trauma, repeated exposure, and psychiatric comorbidity.

## Introduction

In 2014, trauma-associated sleep disorder (TSD) was proposed as a distinct rapid eye movement (REM)-sleep parasomnia, characterized by trauma-related nightmares, hyperactivity, and disruptive nocturnal behaviors (DNB), including gross body movements and abnormal vocalizations^1^. TSD is currently not a clinical diagnosis in the diagnostic classification systems^2,3^. Studies in military and veterans populations have reported high comorbidity between TSD features and PTSD ( ~ 60–70%), insomnia (up to 85–90%), and obstructive sleep apnea ( ~ 45–50%)^1,4^. To date, comparative population-based estimates of TSD comorbidity are lacking.

Sleep disturbance is a core symptom of PTSD, which often persists despite improvement in other PTSD symptoms, leading to an ongoing debate regarding whether TSD symptoms are residual PTSD symptoms, treatment-resistant features, or an independent sleep-related disorder^3,5–7^. Although trauma-related nightmares are a symptom of both TSD and PTSD, TSD is distinct from PTSD in that it is characterized by parasomnia-like dream enactment and DNB, which are not included in the diagnostic criteria for PTSD^4,8,9^. TSD remains an investigational clinical phenotype, and may co-occur with PTSD or persist independently of PTSD^4^. TSD also shares phenotypic similarities with parasomnias, particularly REM sleep behavior, such as dream enactment and loss of normal muscle atonia during REM sleep^4^. However, unlike idiopathic REM sleep behavior disorder, TSD is contingent on trauma exposure and includes symptoms of trauma-related nightmares and hyperarousal, situating the proposed disorder within a trauma-related sleep phenotype, rather than a primary neurodegenerative parasomnia.

Prior research of the proposed TSD has predominantly focused on males in high-stress occupations, particularly military service, finding trauma severity and pre-existing mental health conditions to be risk factors for the disorder^1,10–16^. 12.1% of post‑9/11 veterans have been found to have TASD^17^, but its prevalence in other populations is still largely unknown. Studies show that veterans with PTSD have more sleep-related symptoms than those without PTSD, including DNB^13,18^, insomnia^13,19^, startle awakenings^13^, and fewer arousals from non-REM sleep^20^. However, gaps remain in our understanding of how different types of trauma affect sleep, particularly among women and non-military populations. The current study therefore aims to investigate the prevalence of trauma-associated sleep disturbances (TASD) and its associated factors, within a nationally representative population-based cohort of Icelandic women. We show that TASD is most prevalent among those exposed to interpersonal violence, repeated trauma, and recent life stressors, and is strongly associated with psychiatric comorbidity.

## Methods study population

Participants were women who took part in the baseline assessment of the population-based Stress-And-Gene-Analysis (SAGA) cohort study conducted in 2018-2019. All Icelandic speaking women aged 18–69 years residing in Iceland were eligible and invited to participate (*n* = 104,197). In total, 30,403 women (29.2% of those eligible) participated in the study. The SAGA cohort is broadly representative of the Icelandic female population with respect to age, education, income, and region of residence^21^. For this study, we excluded women with cognitive brain diseases (*n* = 32) and women with missing TASD information (*n* = 718). We excluded cognitive brain diseases to reduce misclassification, as such conditions are frequently associated with abnormal nocturnal motor behaviors^22^ that may overlap with TASD, but arise from different mechanisms. As exposure to life stressor is a prerequisite to TASD, we also excluded women with missing life stressor information (*n* = 1715). The final analytic sample included 27,938 women (Fig. S1).

### Procedure

Women were invited to participate in the study through a phone text message, e-mail, or paper mail. Participation included answering an extensive internet-based questionnaire about trauma history and health, including sleep disturbances. Data were collected from March 2018 to July 2019.

### Measures life stressors

A modified version of the Life Events Checklist for Diagnostic and Statistical Manual of Mental Disorders (Fifth Edition; LEC-5)^23^ was used to assess direct and indirect exposure to life stressors. In addition, other life stressors not included in the LEC-5 were assessed (see Table S1 for the complete list). The worst life stressor was defined as the event or experience that currently bothered the individual the most. Single items were used to assess the frequency of occurrence of the worst life stressor (once; more than once) and the temporal proximity to the event (0–1; 2–4; 5–9; 10–19; ≥20 years). As developmental timing of trauma exposure is associated with differential outcomes across the life course^24,25^, age at first occurrence of the worst life stressor was categorized into developmental periods based on Erik Erikson’s psychosocial stages of human development: infancy/toddlerhood (age 0–3); childhood (age 4–12); adolescence (age 13–19); young adulthood (age 20–39); adulthood (age≥40)^26^. Young adulthood and adulthood were combined to improve statistical robustness and stability of estimates.

Miscarriages and abortions were excluded from TASD analysis because the questionnaire did not collect information on whether these life stressor types met DSM-5 PTSD Criterion A, which is a prerequisite for TASD.

### TASD

Items from two validated self-report instruments were used to assess symptom criteria for TASD. The Pittsburgh Sleep Quality Index Addendum for PTSD (PSQI-A), a seven-item scale, was used to assess DNB that may represent trauma-specific sleep disturbances, including nightmares, abnormal movements, and other DNB^27^. Second, one item was used from the PTSD Checklist for DSM-5 (PCL-5), a 20-item scale which is used to assess past-month symptoms of probable PTSD based on individual’s worst life stressor^28^. Past-month TASD was defined using established symptom criteria, requiring all of the following^1^

1. Exposure to a significant life stressor that meets DSM-5 Criterion A for PTSD^3^;
2. A PSQI-A total score greater than three, indicating clinically relevant trauma-related sleep disturbance ^27^;
3. Presence of trauma-related nightmares, defined as endorsement of nightmares on PCL-5 item two and PSQI-A item three (score ≥ 1)^27,28^; and
4. Presence of DNB, including episodes of terror or vocalizations (PSQI-A item 6 rated ≥ 1) or abnormal motor behaviors, such as kicking, punching, running, or screaming (PSQI-A item 7 rated ≥ 1) ^27^.

### Background information

Demographic information included age, relationship status (married or in relationship; single or widowed), education level (primary; secondary; tertiary level A; tertiary level B), personal monthly income ( < 1200 Euro (EUR); 1201–2400 EUR; 2401–4000 EUR; 4001–5600 EUR; ≥5601 EUR, conversion rates according to the Central Bank of Iceland, February 1, 2018)^29^, and employment status (employed/studying; unemployed, staying at home/maternity leave; on disability; sick leave; retired). Smoking (never; previous smoker; non-daily; daily) and binge drinking in past year (never; less than once a month; monthly; once or more a week) were also assessed.

### Sleep problems

The Pittsburgh Sleep Quality Index (PSQI)^30^, a 19-item self-report scale, was used to assess general sleep problems in the past month. The PSQI global score >10 was used as a cut-off for general sleep problems, in line with previous studies ^31,32^.

### Comorbid mental disorders

The PCL-5 was used to assess probable past month PTSD^28^. Probable PTSD was defined as having a cutoff score of ≥ 33 on the PCL-5 and meeting Criterion A and Criterion B, C, D, and E (items rated ≥ 2 were defined as a symptom reported). The Patient Health Questionnaire (PHQ-9)^33^, a nine-item self-report scale, was used to assess depression symptoms in the past two weeks. The PHQ-9 total scores of <10, 10–19, and, 20–27 represented mild/moderate, moderately severe, and severe depression, respectively. The World Health Organization World Mental Health Composite International Diagnostic Interview (WHO WMH-CIDI) was used to assess the presence or absence of self-harm, suicidal ideation, and suicidal attempts over the past five years^34^. The General Anxiety Disorder scale (GAD-7), a seven-item self-report scale, was used to assess anxiety symptoms in the past two weeks. The GAD-7 total scores of <10, 10–14, and 15–21 represented minimal/mild, moderate, and severe anxiety, respectively ^35^.

### Statistics and reproducibility

This study followed the Strengthening the Reporting of Observational studies in Epidemiology (STROBE) guidelines reporting guideline^36^. All analyses were conducted in R (4.5.2) using RStudio (V.2026.04.1).

**Fig. 1:**
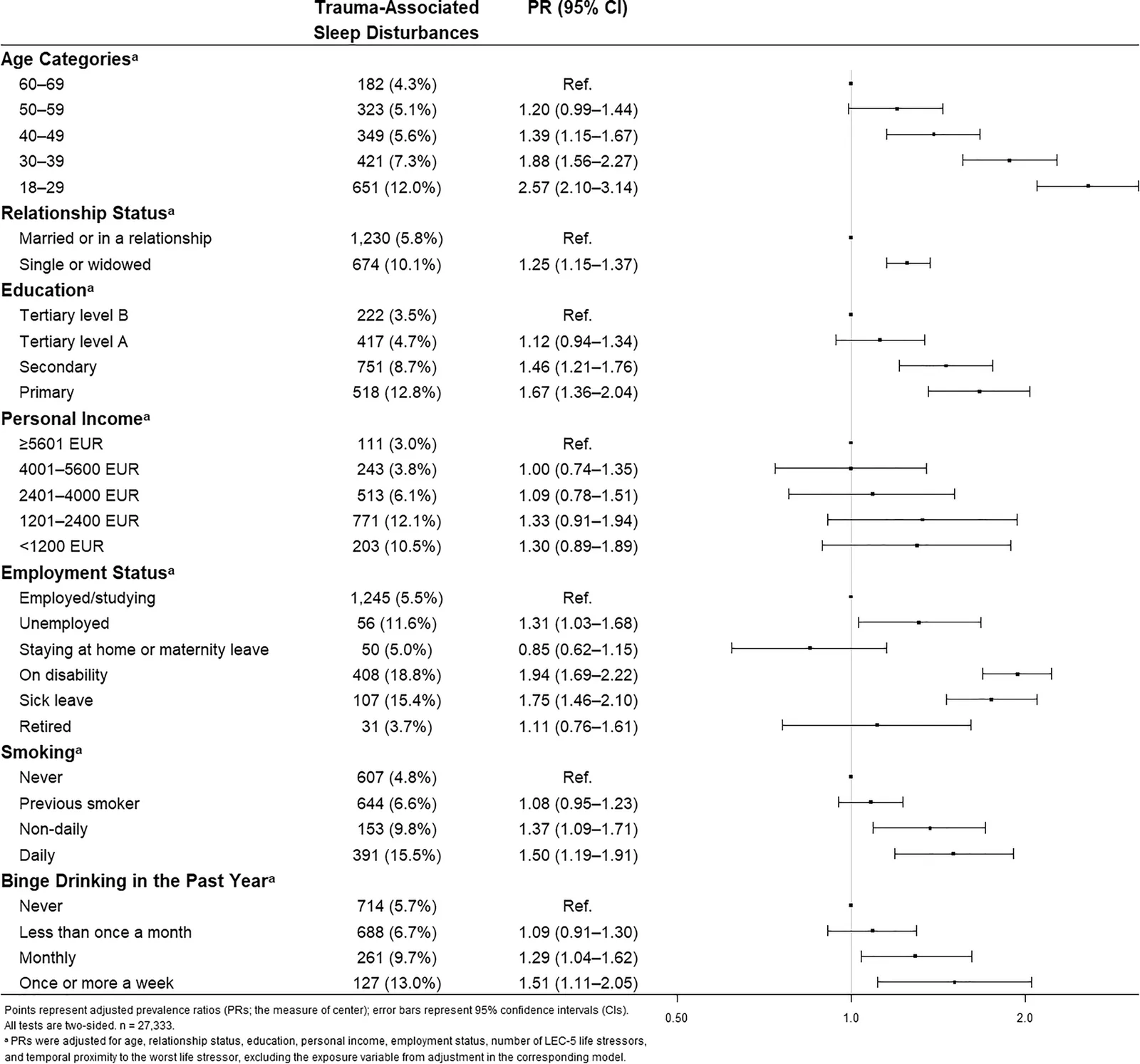
Association of trauma-associated sleep disturbances (TASD) with sociodemographic characteristics and health-related behavior. Note: Estimates are shown on a logarithmic scale and based on pooled imputed data using two-sided tests.

**Fig. 2:**
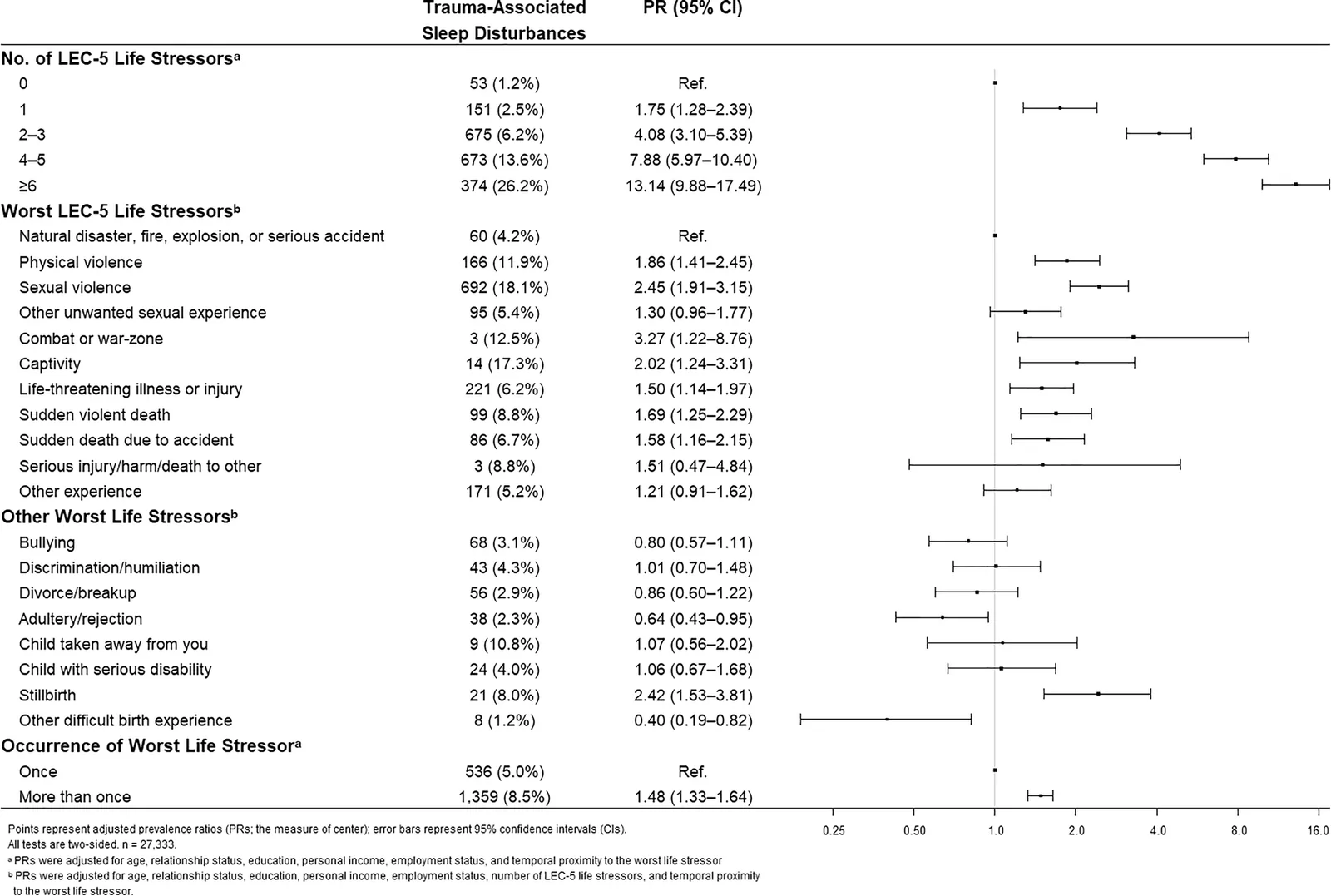
Association of TASD with worst life stressors and occurrence of worst life stressor. Note: Estimates are shown on a logarithmic scale and based on pooled imputed data using two-sided tests.

**Fig. 3:**
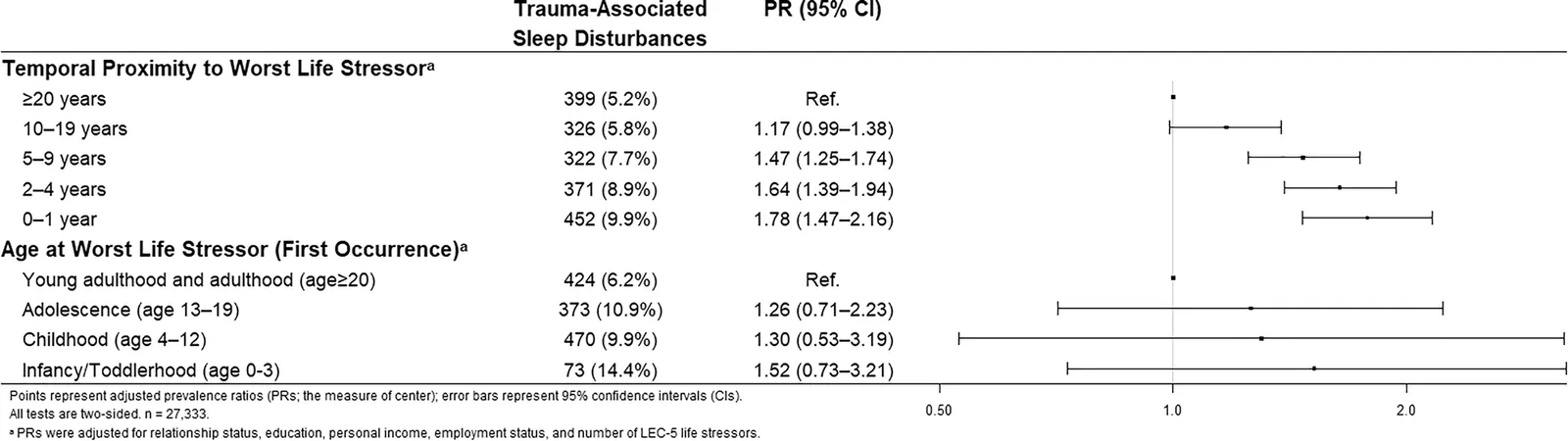
Association of TASD with temporal proximity to worst life stressor and age at the first occurrence of worst life stressor. Note: Estimates are shown on a logarithmic scale and based on pooled imputed data using two-sided tests.

**Fig. 4:**
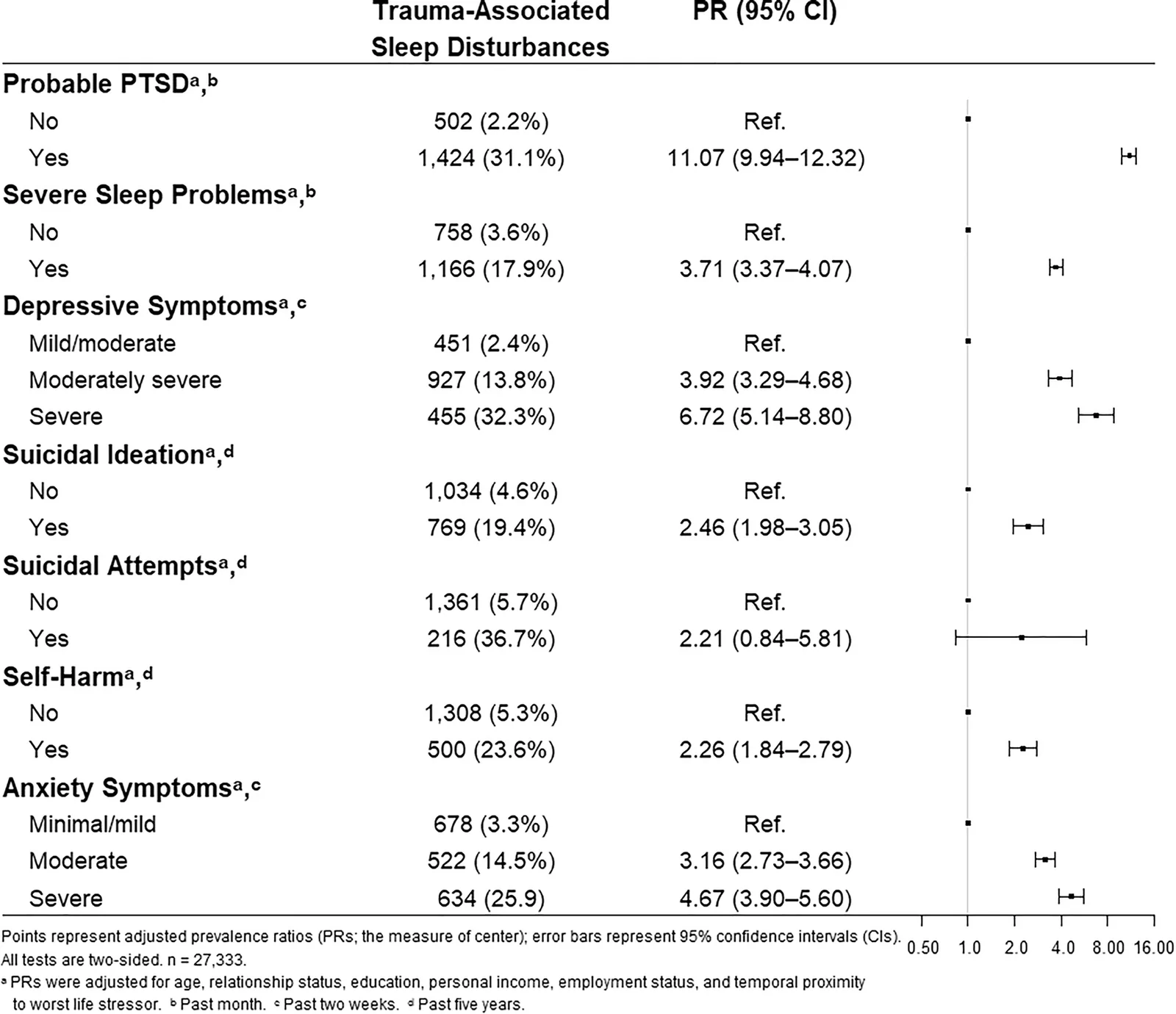
Association of TASD and mental well-being. Note: Estimates are shown on a logarithmic scale and based on pooled imputed data using two-sided tests.

Participants with > 75% missing items on the PSQI-A, PCL-5, PHQ-9, and GAD-7 scales were excluded from the analyses. Those with *≤* 75% missing items were retained and imputed with row imputation. Missing data in the final analytic dataset were addressed using multivariate imputation by chained equations (MICE), with predictive mean matching, generating 20 imputed datasets with 20 iterations^37^. Regression models were fitted within each imputed dataset and pooled to obtain prevalence ratios (PRs) and 95% confidence intervals (CIs). All main regression results presented in Figs. 1–4 are based on pooled estimates from the imputed datasets.

Descriptive statistics were used to summarize participant characteristics. Descriptive statistics presented in Table 1 reflect the analytic sample and were not pooled across imputations. TASD was modeled as a binary outcome. The prevalence of TASD was calculated as the proportion of participants meeting the criteria for TASD within the analytic sample. Women who had not experienced a life stressor (*n* = 605) were excluded from regression models, as exposure to a life stressor is a prerequisite for TASD.

**Table 1:** Demographic characteristics of participants

	Overall
	n = 27,938
**Age in Years, M(SD)**	43.7 (13.6)
**Age Categories, n(%)**	
18–29	5435 (19.5%)
30–39	5728 (20.5%)
40–49	6227 (22.3%)
50–59	6330 (22.7%)
60–69	4218 (15.1%)
**Marital Status, n(%)**	
Married or in a relationship	21,111 (76.0%)
Single or widowed	6679 (24.0%)
Unknown	148
**Education, n(%)**	
Tertiary level B	6320 (22.7%)
Tertiary level A	8873 (31.9%)
Secondary	8606 (30.9%)
Primary	4043 (14.5%)
Unknown	105
**Personal Income, n(%)**	
≥5601 EUR	3749 (14.0%)
4001–5600 EUR	6406 (23.9%)
2401–4000 EUR	8392 (31.3%)
1201–2400 EUR	6363 (23.7%)
<1200 EUR	1929 (7.2%)
Unknown	1099
**Employment Status, n(%)**	
Employed/studying	22,571 (81.3%)
Unemployed	482 (1.7%)
Staying at home/maternity leave	991 (3.6%)
On disability	2166 (7.8%)
Sick leave	696 (2.5%)
Retired	847 (3.1%)
Unknown	185
**No. of LEC-5 Life Stressors**^**a**^**, n(%)**	
0	4529 (16.2%)
1	6097 (21.8%)
2–3	10,920 (39.1%)
4–5	4966 (17.8%)
≥6	1426 (5.1%)
**Temporal Proximity to Worst Life Stressor, n(%)**	
≥20 years	7631 (28.5%)
10–19 years	5614 (21.0%)
5–9 years	4194 (15.7%)
2–4 years	4169 (15.6%)
0–1 year	4570 (17.1%)
Unknown	1155

Modified Poisson regression models with robust (sandwich) variance estimators were used to estimate PRs and 95% CIs^38,39^ for associations between TASD and sociodemographic, trauma-related, and psychiatric factors. Both age-adjusted and multivariable-adjusted models were fitted. Multivariable models were adjusted for age, relationship status, education, personal income, employment status, number of LEC-5 life stressors, and temporal proximity to the worst life stressor, when relevant. Covariates included in multivariable models were selected a priori based on existing literature and theoretical relevance^32,40,41^. Models were fitted using generalized estimating equations via the *geeglm* function^42^, and forest plots were generated using the *forestplot* package ^43^.

When examining the association between worst life stressors and TASD, natural disasters were used as the reference category, as they are relatively common in Iceland and have been associated with low prevalence of trauma-related psychiatric morbidity in population-based samples ^44,45^.

Supplementary Data 5 presents sensitivity analyses comparing PRs from modified Poisson regression models across complete case, available-case (participants with partial data retained without multiple imputation), and pooled multiple-imputation analyses. Supplementary Figs. S2–S5 were generated using the available-case analytic dataset.

Age-stratified analysis were conducted to explore potential variation in observed associations (Supplementary Data 6). Effect modification by age was assessed using Poisson GEE models with interaction terms, and age-stratified PRs were estimated using contrast matrices and generalized linear hypothesis testing.

Approval for the study was granted by the Icelandic Bioethics Committee (NBC nr. 17-238), and the Icelandic Data Protection Authority was notified of the study. All participants provided written informed consent prior to enrollment in the SAGA cohort and could withdraw from the study at any time.

## Results

### Sample characteristics

Among the 27,938 women in the study (mean age 43.7 [SD 13.6] years), TASD prevalence was 6.9%. Most participants were married or in a relationship (76.0%), held university degrees (54.6%), reported medium or high personal income (69.2%), were employed (81.3%), and had experienced 2–3 life stressors according to the LEC-5 (39.1%; Table 1). The most prevalent symptoms reported on the PSQI-A were general nervousness and hot flashes, while DNB were the most infrequent symptoms (Fig. S2).

### Background information and associations with TASD

After adjusting for covariates, we observed the highest prevalence of TASD among women in the youngest age group (adjusted PR [aPR], 2.57; 95% CI, 2.10–3.14), compared to those in the highest age group (60–69 years old; Fig. 1). Additionally, being single or widowed was associated with a higher prevalence of TASD (aPR, 1.25; 95% CI, 1.15–1.37) compared to being married or in a relationship. Furthermore, having secondary and primary education was associated with a 46–67% higher prevalence of TASD when compared to tertiary-level B education. Being unemployed (aPR, 1.31; 95% CI, 1.03–1.68), on disability (aPR, 1.94; 95% CI, 1.69–2.22), or on sick leave (aPR, 1.75; 95% CI, 1.46–2.10) was associated with a higher prevalence of TASD, compared to being employed or studying. Daily smoking (aPR, 1.50; 95% CI, 1.19–1.91), non-daily smoking (aPR, 1.37; 95% CI, 1.09–1.71), as well as monthly (aPRs, 1.29; 95% CI, 1.04–1.62) and weekly (aPRs, 1.51; 95% CI, 1.11–2.05) binge drinking, were associated with a higher prevalence of TASD compared to non-smoking and non-binge drinking. No association was found between TASD and personal income (Fig. 1). A stratified analysis by age groups (Supplementary Data 6) revealed significant interactions between age and education, employment status, worst life stressor type, and how often the worst life stressor occurred (p <0.05).

### Life stressors and TASD

We observed a notable increase in TASD prevalence with the number of LEC-5 life stressors encountered (Fig. 2 and S3). Notably, certain life stressors exhibited particularly strong associations with TASD. With exposure to natural disasters as reference, we observed highest prevalence of TASD when the worst life stressor was physical violence (aPR, 1.86; 95% CI, 1.41–2.45), sexual violence (aPR, 2.45; 95% CI, 1.91–3.15), combat/war-zone (aPR, 3.27; 95% CI, 1.22–8.76), captivity (aPR, 2.02; 95% CI, 1.24–3.31), life-threatening illness or injury (aPR, 1.50; 95% CI, 1.14–1.97), sudden violent death (aPR, 1.69; 95% CI, 1.25–2.29), accidental sudden death (aPR, 1.58; 95% CI, 1.16–2.15), and stillbirth (aPR, 2.42; 95% CI, 1.53–3.81). Conversely, adultery/rejection and other difficult birth experiences were found to be associated with a lower prevalence of TASD. Other life stressors such as bullying, discrimination, and divorce were non-significant.

Women who experienced their worst life stressor more than once reported a 48% higher prevalence of TASD compared to those who experienced it once (Fig. 2). Years since worst life stressor showed a graded association with TASD, with women exposed within a year displaying the highest prevalence of TASD and women exposed more than 20 years ago the lowest (Fig. 3). Stratification for age at study entry revealed that this graded association was somewhat evident in all age groups; however, the prevalence was highest among women aged 18–29 years (Figure S4). No association was found between the prevalence of TASD and age at the first occurrence of the worst life stressor.

### Psychiatric comorbidity

After adjusting for all covariates, TASD was found to be strongly associated with probable PTSD (aPR, 11.07; 95% CI, 9.94–12.32; Fig. 4). Further analysis revealed that 74% (*n* = 1424/1926) of individuals with TASD also had probable PTSD. Additionally, individuals with general sleep problems had a higher prevalence of TASD (aPR, 3.71; 95% CI, 3.37–4.07), compared to those without general sleep problems. Examining the overlapping prevalence of TASD, probable PTSD, and general sleep problems, we found that 3.5% of the total sample exhibited all three conditions, while only 1.1% had TASD without the other two (Fig. S5). Moderately severe (aPR, 3.92; 95% CI, 3.29–4.68) or severe (aPR, 6.72; 95% CI, 5.14–8.80) depressive symptoms were associated with a higher prevalence of TASD compared to mild or moderate depressive symptoms. Suicidal ideation (aPR, 2.46; 95% CI, 1.98–3.05) and self-harm (aPR, 2.26; 95% CI, 1.84–2.79) in the past five years were also linked with TASD. Additionally, moderate (aPR, 3.16; 95% CI, 2.73–3.66) and severe (aPR, 4.67; 95% CI, 3.90–5.60) anxiety symptoms were associated with TASD compared to minimal or mild symptoms.

## Discussion

In this large cohort of adult women, we established a population prevalence of TASD of 6.9% which was considerably higher among young women. Exposure to physical and sexual violence, combat, and stillbirth was associated with markedly higher prevalence of TASD than other life stressors. Close temporal proximity to the worst life stressor and repeated events were also associated with higher prevalence of TASD, particularly among young women. Finally, TASD was strongly associated with current symptoms of PTSD, depression, and anxiety.

Our investigation revealed that 6.9% of women met criteria for TASD in the past month, marking the first examination of TASD prevalence in a general population sample since its introduction in 2014^1^. This estimate is lower than the 12.1% TASD prevalence reported in a cohort of 3618 veterans, where men and women were similarly affected^17^. Among women in our study exposed to war/combat we observed a 12.5% prevalence. However, as few women report war/combat exposure as their worst life stressor in this cohort (*n* = 24), these results warrant cautious interpretation, as prevalence estimates of TASD in this subgroup are likely imprecise. Previous studies have found combat exposure to be associated with frequent nightmares, nocturnal behaviors, sleep disturbances, and altered REM sleep^13,18–20,46^. Terror or screaming during sleep and dream enactment were relatively infrequent in our study, (11.5% and 8.7%, respectively) but higher than the 5% prevalence of dream enactment found in a general Norwegian sample, involving both genders ^47^.

Our study revealed a strong link between TASD and the number of life stressors, with prevalence increasing progressively with each additional stressor experienced. Previous research suggests that higher cumulative count of traumatic experiences is associated with TASD and an increased risk of PTSD, nightmares, and insomnia^17,41,48^. Interpersonal trauma, especially exposure to sexual violence, was associated with high prevalence of TASD in our study. Such trauma can profoundly impact physical and mental well-being, particularly if the assault occurs during sleeping hours, undermining a sense of safety and leading to sleep avoidance due to fear of nightmares or loss of control ^49,50^.

Our study also identified a high TASD prevalence among women who had experienced stillbirth, aligning with research indicating significant emotional, psychological, and physiological impacts of stillbirth on women, including depression, anxiety, and PTSD^51^. Life-threatening illness, injury, and witnessing a sudden death were also found to be associated with higher prevalence of TASD. In female U.S. veterans with TASD, the most common traumatic events associated with TASD were combat (41.4%), illness (11.1%), and civilian/non-military physical attacks (10.1%)^17^. While research on TSD among survivors of life-threatening illnesses is limited, PTSD has been reported in 12–25% of individuals after acute medical events like heart attacks, strokes, and cancer^52^. These findings highlight the nuanced connection between distinct trauma types and TASD development, suggesting that TASD onset is linked to more severe and personal traumatic experiences^9^. Stressors such as adultery or rejection by a spouse and other difficult birth experiences were associated with low TASD prevalence. The interpretation of these findings is challenging, as this is the first study to examine these stressors in relation to TASD, and the heterogeneity of these categories and limited contextual information may have influenced the observed associations.

Our results also revealed that repeated exposure to the worst life stressor and close temporal proximity to the worst life stressor were associated with higher TASD prevalence, consistent with PTSD studies^53^. In line with previous research on 823 US female veterans^17^, we found close temporal proximity to the worst life stressor to be associated with higher TASD prevalence, particularly among women younger than 30. Sexual abuse, which is prevalent as the worst traumatic event in this cohort, likely contributes to this trend, as it occurs most frequently during the teenage years and young adulthood^54^. The severity of nightmares and DNB tends to be most pronounced in the wake of the trauma, occurring frequently in the weeks and months immediately following the event^1,10^. While these symptoms may persist over time, their frequency and intensity typically diminish^12,20^. Stratification by age at study entry indicated that TASD prevalence was highest among women aged 18–29 years, with the graded association between temporal proximity to the worst life stressor and TASD evident across age groups. In contrast, no clear association was observed between TASD prevalence and age at first occurrence of the worst life stressor. Taken together, these findings suggest that current age and recency of trauma exposure may be more salient for TASD prevalence than the developmental timing of first exposure. While prior research indicates that early-life trauma may confer long-term vulnerability in stress regulation and sleep-related functioning (e.g., persistent nightmares and hyperarousal)^55^, the extent to which developmental timing specifically influences TASD remains unclear.

TASD prevalence was strongly associated with comorbid psychiatric symptoms, particularly probable PTSD, with a 74% prevalence among TASD women, aligning with other studies’ 50–76% range^1,17^. The overall prevalence of probable PTSD in the SAGA cohort has previously been reported as 15.9%^44^. The association between TSD and PTSD is well-documented in the literature as these are both conditions triggered by exposure to traumatic events and often co-occur^1,4,16,56^. The overlapping symptoms, particularly those related to re-experiencing the event, such as recurrent distressing dreams and flashbacks, underscore the interconnected nature of these conditions^1^. Despite the well-documented correlation, further research is crucial to understand the mechanisms underlying this comorbidity and to develop effective interventions aimed at addressing the symptoms of both PTSD and TSD. Furthermore, our study found that women with depressive and anxiety symptoms, suicidal ideation and self-harm had higher prevalence of TASD. This is in line with the findings of a study of post-9/11 veterans where those with TSD had a higher prevalence of PTSD, major depressive disorder, and suicide ideation, than those without TSD^17^. Women with general sleep problems in our study, as measured by the PSQI, had a higher prevalence of TASD, highlighting the interconnectedness of broad sleep symptoms and trauma^46^. It is unclear whether these general sleep problems preceded trauma, but pre-existing sleep problems may influence trauma outcomes, as seen in veterans with pre-war nightmares having higher PTSD risk post-war^57^. The current study marks the first exploration of comorbidity of TASD, probable PTSD, and general sleep problems, with 3.5% of the sample exhibiting all three conditions. This study also indicates that a subset of individuals met criteria for TASD without having probable PTSD, suggesting that TASD may represent a unique trauma-related sleep phenotype that is not captured by PTSD diagnostic criteria. Trauma-related sleep disruption may therefore occur independently of PTSD symptomatology. The presence of TASD in the absence of probable PTSD suggests that trauma exposure can affect sleep-related processes, such as REM dysregulation or nocturnal hyperarousal, without broader PTSD symptomatology. Individuals with TASD may benefit from targeted sleep-focused interventions addressing trauma-related nightmares and DNB. Longitudinal research is needed to clarify mechanisms and potential prevention implications.

There are two notable strengths in this study. Firstly, a key strength of this study is the use of data from the large, nationwide SAGA cohort, which allows investigation of TASD and its associated factors in a population-based sample of adult women^21^. Secondly, this study represents the largest cohort to date to assess TASD and factors associated with TASD among women in the general population. To our knowledge, no other large cohort studies have explored TASD in this demographic. Despite these notable strengths, several limitations should be considered. First, the cross-sectional design precludes conclusions about the directionality of associations between TASD and the assessed comorbidities. Second, the study relies exclusively on self-reported measures of trauma exposure and sleep-related symptoms, which may be subject to recall or reporting bias. Third, polysomnographic assessment was not available, precluding evaluation of REM sleep without atonia, which is part of the proposed diagnostic criteria for TSD. However, prior clinical work has noted that DNB and trauma-related nightmares are frequently reported by trauma survivors and their bed partners but may be difficult to document in laboratory sleep settings, complicating objective verification even in clinical studies^9^. Fourth, we adjusted our analyses for several potential confounding factors; however, it is possible that some of these variables, such as alcohol use and smoking, are in certain cases at least partially consequences of TASD. Fifth, although the underlying cohort is representative of Icelandic women aged 18 to 69 years old with respect to key sociodemographic characteristics, the participation rate was relatively modest (29%). Selection bias related to trauma exposure or mental health cannot be excluded. Prevalence estimates may therefore have been influenced in either direction, toward lower TASD prevalence if women with more severe trauma exposure or mental illness were less likely to participate, or toward higher prevalence if trauma-exposed women were more motivated to take part. Finally, as the study focused on women living in a Nordic welfare state, the findings may not be directly generalizable to other populations. Large longitudinal studies across more diverse settings, including men, adolescents, and children, are therefore needed to further advance understanding of TASD.

In conclusion, this study provides critical insights into factors influencing the prevalence of TASD and its psychiatric comorbidities in a nationally representative population of women. Exposure to sexual violence, physical violence, and stillbirth was associated with high prevalence of TASD. Repeated trauma exposure and temporal proximity to the worst life stressor emerged as risk factors for TASD. Our results support trauma-informed screening and targeted sleep-focused interventions for women exposed to high-impact stressors.

## Supplementary information

Supplementary Materials

Description of Additional Supplementary files

Supplementary Data 1.

Supplementary Data 2.

Supplementary Data 3.

Supplementary Data 4.

Supplementary Data 5.

Supplementary Data 6.

## Data Availability

The individual-level data underlying this study were generated as part of the SAGA cohort and are subject to ethical approval and data protection regulations in Iceland. Researchers seeking access to the data for replication or secondary analyses may apply through the SAGA data access portal on (www.afallasaga.is). Requests are reviewed by the SAGA data governance committee in accordance with Icelandic data protection laws. The data are securely stored on protected servers in Iceland. The source data underlying Figs. 1–4 are provided in Supplementary Data 1, Supplementary Data 2, Supplementary Data 3, and Supplementary Data 4, respectively. Supplementary Data 5 contains the sensitivity analyses comparing complete-case, available-case, and pooled multiple-imputation results, and Supplementary Data 6 contains the age-stratified analyses.

## Code availability

The custom R code used to preprocess the data and to perform the regression analyses underlying this study has been deposited in a public GitHub repository and is available at https://github.com/annaunnars/TASD.
