# Supplementary material for: Trauma-associated sleep disturbances among women in a nationwide study
Source: Commun Med (Lond). 2026 Jun 17;6:534. doi: 10.1038/s43856-026-01698-0 (PMC13669314; doi:10.1038/s43856-026-01698-0)
Supplement: Supplementary file 1 [file 43856_2026_1698_MOESM1_ESM.pdf]

Supplementary Materials

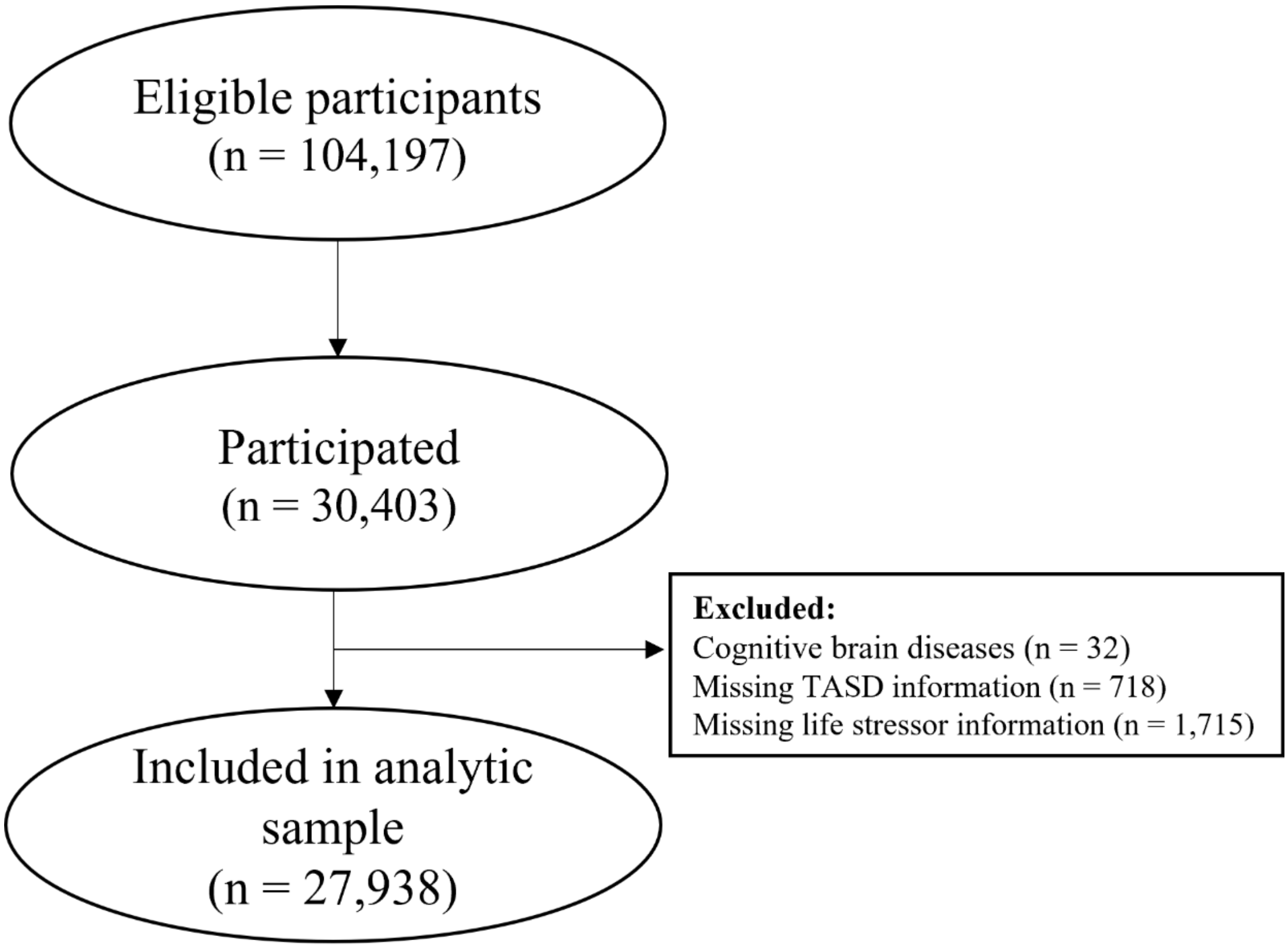

Figure S1. Flowchart of women participating in the study.

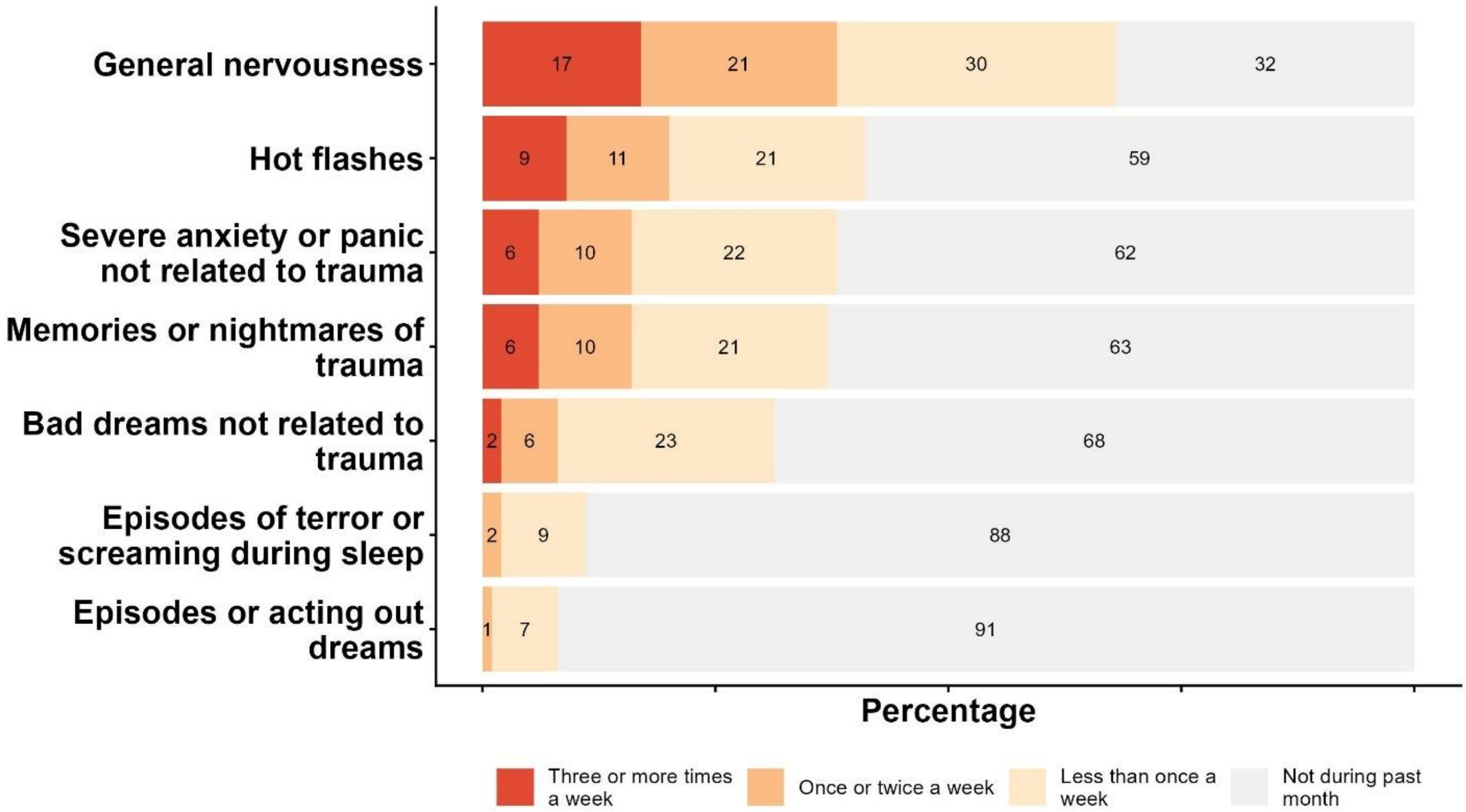

Figure S2. Past-month prevalence of trauma-related and non-trauma-related sleep symptoms measured by the PSQI-A. Note: Values below 1% are not displayed. PSQI-A = The Pittsburgh Sleep Quality Index Addendum for PTSD, n = 27,938.

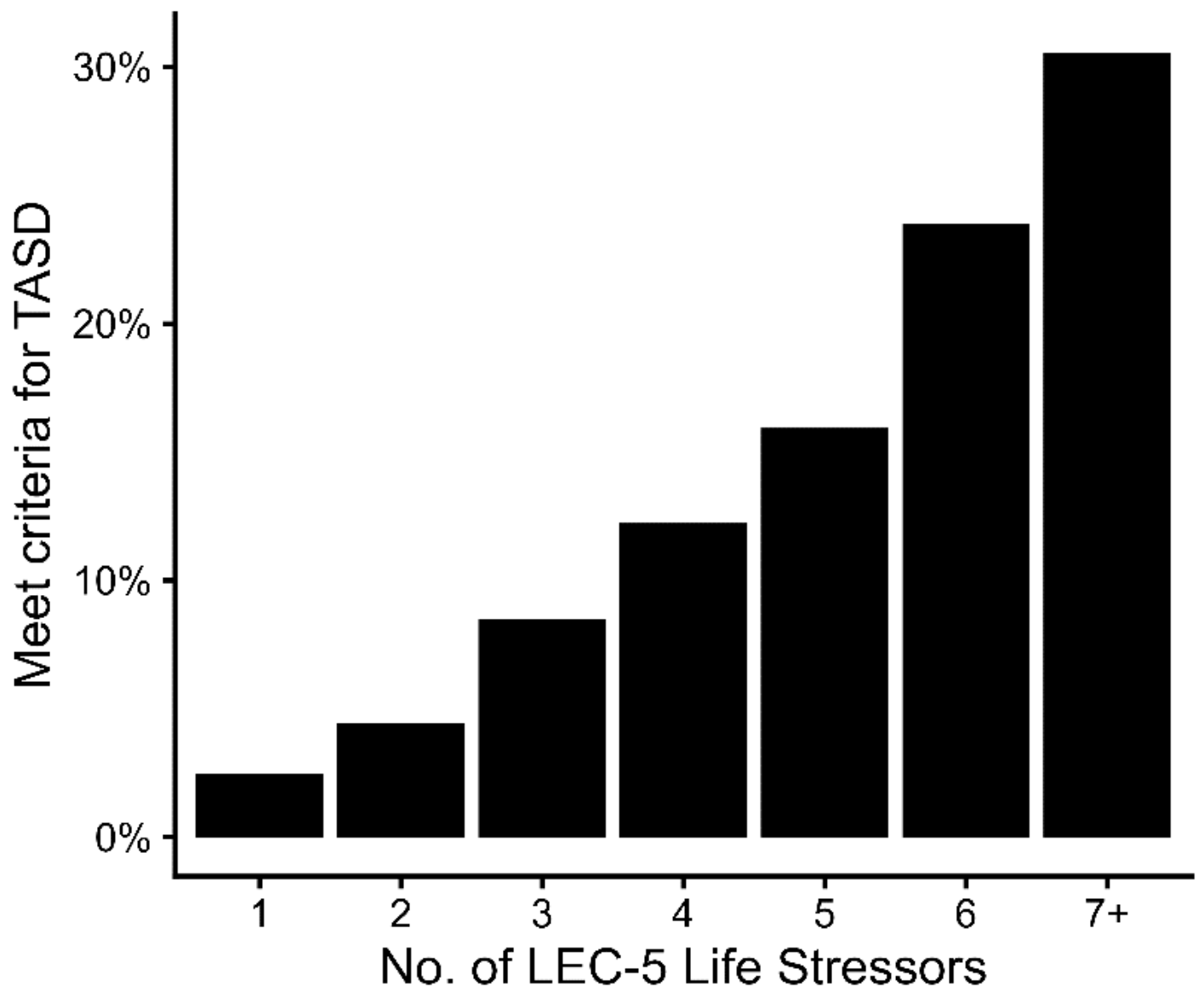

Figure S3. Association between number of LEC-5 life stressors and TASD among individuals with ≥1 life stressor (n = 23,409).

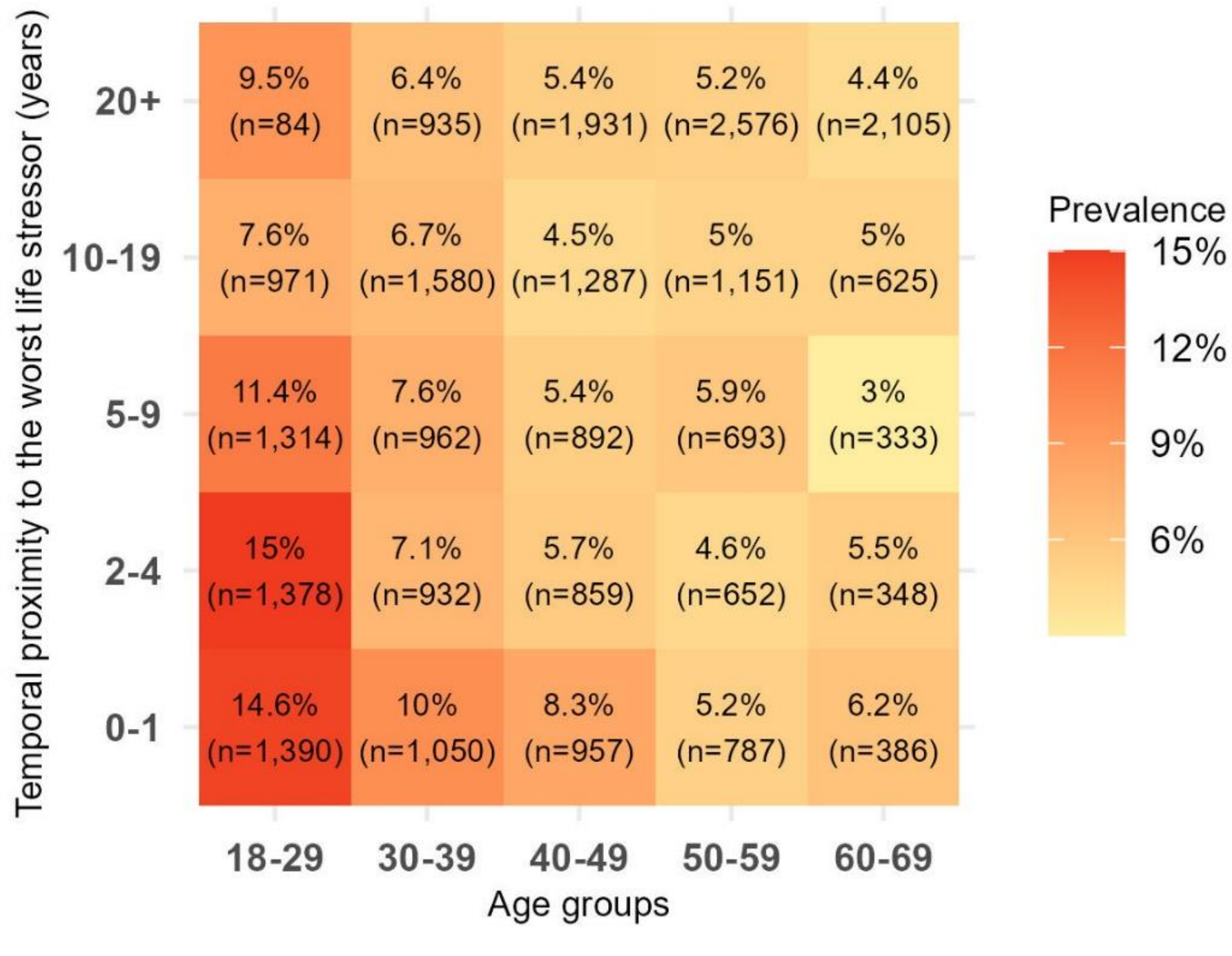

Figure S4. Heatmap of TASD prevalence across age groups and temporal proximity to worst life stressor. Note: Analyses are restricted to participants with a reported worst life stressor and known temporal proximity to that stressor, n = 26,178.

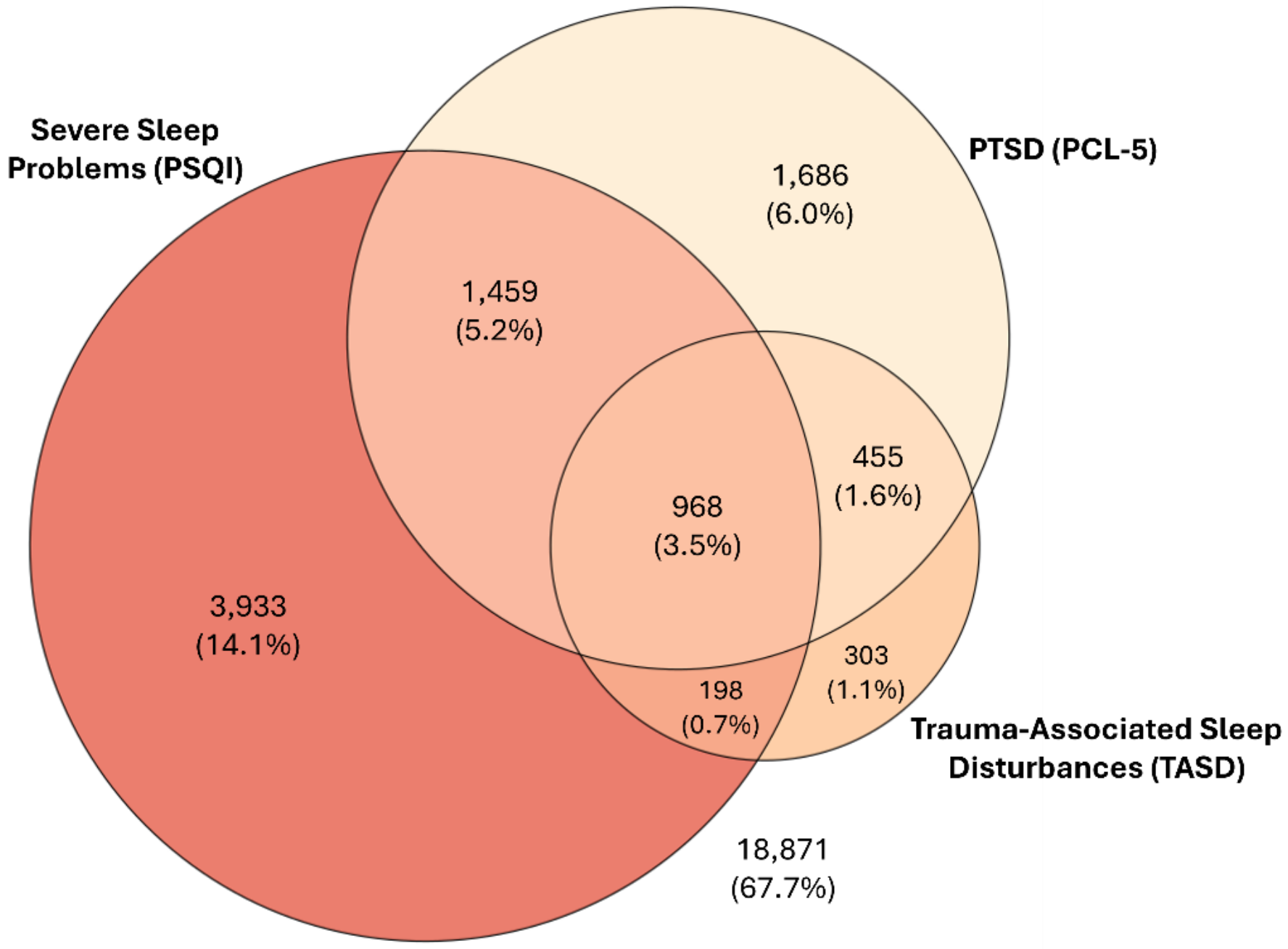

Figure S5. Overlap between TASD, severe sleep problems measured by the PSQI, and probable PTSD measured by the PCL-5. Note: Area sizes are proportional to the number of cases. Participants with missing data on any measure were excluded, n = 27,873.

Table S1 – Worst life stressor items

No.	Life stressor	Further description
**LEC-5***		
1.	Natural disaster, fire, explosion, or serious accident	Natural disaster (e.g., flood, hurricane, tornado, earthquake), fire, explosion, transportation accident (e.g., car accident, boat accident, train wreck, plane crash), or serious accident at work, home, or during recreational activity
2.	Physical violence	Physical assault (e.g., being attacked, hit, slapped, kicked, beaten up) or assault with a weapon (e.g., being shot, stabbed, threatened with a knife, gun, bomb)
3.	Sexual violence	Sexual assault (e.g., rape, attempted rape, made to perform any type of sexual act through force or threat of harm
4.	Other unwanted sexual experience	Other unwanted or uncomfortable sexual experience
5.	Combat, or war-zone	Combat, or exposure to a war-zone (in the military or as a civilian)
6.	Captivity	Loss of freedom (e.g., kidnapping, abduction, taken hostage, prisoner of war)
7.	Life-threatening illness or injury	
8.	Sudden violent death	E.g., murder, suicide
9.	Sudden death due to accident	
10.	Serious injury/harm/death to other	Serious injury, harm or death that you caused someone else
**Other life stressors**		
11.	Bullying	Repeated reprehensible behaviour of a person or a group, intended to frighten, hurt, or intimidate others
12.	Discrimination/humiliation	Discrimination (e.g., prejudice because of race or sexual orientation), public humiliation on social media or in the press, or other humiliating experience
13.	Divorce/breakup	
14.	Adultery/rejection	Adultery/rejection by spouse
15.	Child taken away from you	
16.	Child with serious disability	Having a child with a serious physical or mental disability (e.g., impaired development, birth defect, impaired vision, hearing, or walking)
17.	Stillbirth	
18.	Other difficult birth experience	
19.	Other experience	Open ended question on other stressful/traumatizing experience.

* LEC-5 = Life Events Checklist for Diagnostic and Statistical Manual of Mental Disorders (Fifth Edition)
