# Supplementary material for: Trauma-associated sleep disturbances among women in a nationwide study
Source: Commun Med (Lond). 2026 Jun 17;6:534. doi: 10.1038/s43856-026-01698-0 (PMC13669314; doi:10.1038/s43856-026-01698-0)
Supplement: Supplementary file 2 [file 43856_2026_1698_MOESM2_ESM.docx]

**Description of Additional Supplementary Files**

File name: Supplementary Data 1

Description: Aggregated source values plotted in Figure 1 (Association of TASD with sociodemographic characteristics and health-related behavior). Includes category counts, percentages, adjusted prevalence ratios, and 95% confidence intervals for age, relationship status, education, personal income, employment status, smoking, and binge drinking.

File name: Supplementary Data 2

Description: Aggregated source values plotted in Figure 2 (Association of TASD with worst life stressors and occurrence of worst life stressor). Includes category counts, percentages, adjusted prevalence ratios, and 95% confidence intervals for number of LEC-5 life stressors, worst LEC-5 life stressor, other worst life stressors, and occurrence of the worst life stressor.

File name: Supplementary Data 3

Description: Aggregated source values plotted in Figure 3 (Association of TASD with temporal proximity to worst life stressor and age at first occurrence of worst life stressor). Includes category counts, percentages, adjusted prevalence ratios, and 95% confidence intervals.

File name: Supplementary Data 4

Description: Aggregated source values plotted in Figure 4 (Association of TASD and mental well-being). Includes category counts, percentages, adjusted prevalence ratios, and 95% confidence intervals for probable PTSD, severe sleep problems, depressive symptoms, suicidal ideation, suicidal attempts, self-harm, and anxiety symptoms.

File name: Supplementary Data 5

Description: Sensitivity analyses across complete-case, available-case, and pooled multiple imputation analyses.

Comparison of prevalence ratios from modified Poisson regression models across three analytic approaches: complete-case analysis, available-case analysis (with "Unknown" categories retained), and pooled multiple imputation analysis (m = 20). Includes crude and adjusted estimates for all exposures examined in the main analysis.

File name: Supplementary data 6

Description: Age-stratified analyses of associations between TASD and study covariates.

Stratified analyses by age group (18–39, 40–59, 60–69 years) of the association between TASD and sociodemographic characteristics, health-related behaviors, trauma-related outcomes, and psychopathologies, using complete cases (n = 20,464) with multivariate adjustment. Effect modification by age group is expressed as p-values from likelihood ratio tests on the exposure × age-group interaction term.
